# Smoking, cessation and expenditure in low income Chinese: cross sectional survey

**DOI:** 10.1186/1471-2458-7-29

**Published:** 2007-03-04

**Authors:** Therese Hesketh, Li Lu, Ye Xue Jun, Wang Hong Mei

**Affiliations:** 1 Centre for International Child Health, Institute of Child Health, University College London, 30 Guilford St, London WC1 N1EH, UK; 2 Institute of Family and Social Medicine, Zhejiang University, Yan An Lu, Hangzhou 310006, PR China

## Abstract

**Background:**

This study was carried-out to explore smoking behaviour and smoking expenditure among low income workers in Eastern China to inform tobacco control policy.

**Methods:**

A self-completion questionnaire was administered to 1958 urban workers, 1909 rural workers and 3248 migrant workers in Zhejiang Province, Eastern China in 2004.

**Results:**

Overall 54% of the men and 1.8% of all women were current smokers (at least 1 cigarette per day). Smoking was *least *common in migrant men (51%), compared with 58% of urban workers and 64% rural inhabitants (P < 0.0001). Forty-nine percent of rural males smoke more than 10 cigarettes/day, and 22% over 20/day. The prevalence of smoking increased with age. Overall 9% of the males had successfully quit smoking. Reasons for quitting were to prevent future illness (58%), current illness (31%), family pressures (20%) and financial considerations (20%). Thirteen percent of current smokers had ever tried to quit (cessation for at least one week) while 22% intended to quit, with migrants most likely to intend to quit. Almost all (96%) were aware that smoking was harmful to health, though only 25% were aware of the dangers of passive smoking. A mean of 11% of personal monthly income is spent on smoking rising to a mean of 15.4% in rural smokers. This expenditure was found to have major opportunity costs, including in terms of healthcare access.

**Conclusion:**

The prevalence of smoking and successful quitting suggest that smoking prevalence in low income groups in Eastern China may have peaked. Tobacco control should focus on support for quitters, on workplace/public place smoking restrictions and should develop specific programmes in rural areas. Health education messages should emphasise the opportunity costs of smoking and the dangers of passive smoking.

## Background

Although smoking prevalence is decreasing in most countries, there is a widening gap in smoking prevalence between socioeconomic groups: the poor and less well-educated are now more likely to smoke, they smoke more cigarettes and are less likely to quit than the wealthier and better educated [1,2]. In China only very recently has evidence for such a gap started to emerge [3,4]. China makes a huge contribution to the global burden of disease from tobacco[5]. It is the largest producer and consumer of cigarettes in the world and home to more smokers than in all developed countries combined [6,7]. Few Chinese women smoke, but with a smoking prevalence in men of around 60% [8-11] it is estimated that around one third of all Chinese men will die prematurely of a smoking-related disorder [12,13].

With disproportionate numbers of smokers coming from low income groups this raises specific concerns. Not only do the costs of smoking represent a higher proportion of income for poor smokers, but the overwhelming majority of the poor are uninsured and rely on out-of-pocket payments for healthcare, so that expenditure on cigarettes may also have opportunity costs for access to healthcare [14]. This clearly has implications for the health of poor smokers and their families. Smokers themselves are therefore doubly vulnerable to ill-health.

To control tobacco consumption in China is a massive public health challenge and there is increasing awareness that tobacco control measures should particularly target low income groups. This study was carried out to help inform tobacco control measures and to provide baseline measures for interventions in the eastern province of Zhejiang. It aimed to quantify and explore smoking behaviours, knowledge and expenditure in three distinct unskilled low income groups: urban workers, rural peasants and rural-urban migrants. While there are studies of smoking prevalence in rural populations [4,9] there is little information about migrant workers and poor urban populations[11]. Migrant workers are of particular interest because of their large and increasing numbers: 120 million across the country in 2000, with predictions of 160 million or 25% of the working population by 2010[15]. They are also thought to have high levels of stress, resulting from loss of accustomed social networks, insecurity, poor living conditions, and marginalisation in urban communities[16]. In other populations this has been shown to lead to a greater tendency to risk behaviours, including smoking [17].

## Methods

The study was carried-out from June to December 2004 in Hangzhou, the provincial capital, and two rural townships in Zhejiang Province. Hangzhou has a population of 6.2 million with a migrant population of around one million, and is one of the boom cities of the East [18]. The study was carried-out in two of the eight districts of the city, randomly selected to represent suburban (Xiaoshan) and inner-city (Xihu). In each district a list of workunits employing at least 30 workers and providing unskilled employment for both migrants and local people was drawn-up, giving 31 in Xiaoshan and 42 in Xihu. A sample of 50% of these workunits was selected to be representative of the major occupations of migrant workers in Hangzhou. For example, in Xihu 16 of 28 workunits were involved in manufacturing so, the first eight factories on the list drawn-up were approached. In total sixteen workunits in Xiaoshan and 21 in Xihu were asked to participate and none refused. Migrants were defined as individuals who hold rural household registration (*hukou*) and who had been working in Hangzhou for at least three months and up to ten years. In China by law individuals must hold a *hukou*, which ensures certain citizen's rights, including access to education and health care, in the place where the hukou is held. With very few exceptions the household registration stays at the point of departure and so migrants are classified as temporary, irrespective of their length of stay and most eventually return to their rural area [19]. In each workunit all workers (migrant and urban) present on the day were recruited into the study. This led to an almost 3 to 2 ratio of migrants to urban workers.

Rural sampling took place after the migrant data were obtained, since the aim was to include areas from which a large number of the migrants originated. Migrants originated from 27 of the 33 Chinese provinces By far the largest single group (21%) were from Western Zhejiang, so this area was selected to provide the rural sample. Two of the 12 counties in Western Zhejiang were randomly selected and three villages in each of the counties were in turn randomly selected. Households were then randomly sampled from lists held by village authorities to achieve a sample size of approximately 300 respondents per village or a total of 1800. Working adults aged 15 to 52 (to match the age range in the migrant and urban samples) resident in the selected households on the day of the survey were included.

Respondents completed a questionnaire, developed specifically for the study and which included open and closed questions covering:

1)socio-demographic characteristics, 2) lifestyles, including income, 3) smoking knowledge, behaviours and expenditure. Key questions on smoking behaviour were drawn from those used in the national survey of 1996 [20], but additional questions were added to explore smoking behaviour and in particular smoking cessation in more depth. The questionnaire was piloted among a representative sample of 50 respondents across the three groups and was amended prior to finalisation. Questionnaires were completed in the respective workunits in Hangzhou and in respondents' homes in the villages. In both settings questionnaires were completed in the same way, self-administered without conferring with colleagues or family members. But research assistants were on-hand to help with any queries.

Anonymity and confidentiality were assured. Approvals for the study were obtained from the Ethics Committee of the Institute of Child Health, University College London and Zhejiang Bureau of Public Health.

### Analysis

Because of the importance of the dose-response relationship between quantity of cigarettes smoked and health outcomes [21] we created three categories of smoking for analysis purposes: 1)current, defined as smoking at least one cigarette per day (this corresponds with the definition of current smoking used in the 1996 National Survey) 2) moderate, 10–19 cigarettes per day and 3) heavy, 20 or more cigarettes per day. Quitting was defined as having previously smoked, but no longer smoking for at least one month. Attempts at quitting were defined as smoking cessation for at least one week. We used ANOVA to calculate the differences in the means, Pearson Chi-Squared to evaluate the association between smoking status and other variables and multinomial logistic regression to control for the key variables: age and residence. Because of the small numbers of female smokers in each subgroup most of the analysis was carried-out on the men only. Sociodemographic variables were dichotomised for analysis purposes: age to less than 30 and more than 30, education to low (completion of middle school or less) and high(completion of high school or more) and personal income to less than 900 RMB and more than 900 RMB per month.

## Results

### Sociodemographic characteristics

Complete data were obtained from 1958 urban workers, 1909 rural workers and 3248 migrant workers. The response rate was over 99% for both urban and migrant workers (time was allocated by the workunits to allow workers to participate) with a 92% overall response rate in the rural samples. Their sociodemographic characteristics by gender are shown in Table 1. Migrant workers were the youngest of the three groups with rural workers the oldest. (P < 0.0001) As expected urban workers had the highest levels of education, migrants second, with rural residents the least well-educated.(P < 0.0001) Urban workers earned more than migrants, with rural workers the least. The male:female income differential was consistent across the three groups.

**Table 1:** Sociodemographic characteristics of the sample by residence status and gender n(%)

		Urban		Rural		Migrant	
		M	F	M	F	M	F
		N = 843	n = 1115	n = 749	n = 1160	n = 2198	n = 1050

Age in years	Mean (SD)	36 (12.1)	34 (8.8)	39 (13.4)	35 (11.9)	29 (8.3)	25 (6.8)

Education	Primary	88 (11)	93 (8.4)	378 (50)	915 (79)	253 (11)	126 (12)
	Middle	278 (33)	425 (38)	250 (33)	187 (16)	1259 (58)	683 (65)
	High	309 (37)	416 (37)	106 (14)	47 (4.1)	616 (28)	220 (21)
	Tertiary	168 (18)	170 (15)	15 (2)	11 (0.9)	66 (2.7)	21 (2.0)

Marital status	Single	172 (20)	134 (12)	85 (11)	38 (3.3)	934 (43)	557 (53)
	Married	659 (78)	946 (85)	624 (83)	1041 (89)	1203 (55)	484 (46)
	Other	12 (1.4)	35 (2.9)	40 (5.3)	81 (7)	61 (2.5)	9 (0.9)

Child/ren	Yes	617 (73)	920 (83)	642 (86)	1101 (95)	1105 (50)	399 (38)

Occupation	Agriculture	30 (3.5)	35 (3.1)	584 (78)	731 (63)	2 (0.2)	0
	Factory**	420 (49)	460 (41)	16 (2)	58 (5)	903 (39)	617 (59)
	Construction	22 (2.6)	5 (0.5)	0	0	599 (29)	2 (0.2)
	Hotels/bars	101 (12)	280 (25)	13 (2)	70 (6)	549 (20)	229 (22)
	Retail (shops)	85 (10)	113 (10)	14 (2)	0	131 (6)	11 (1)
	Transport	0	0	7 (1)	0	14 (1.2)	3 (0.3)
	Domestic service	16 (2)	2 (0.2)	0	35 (3)	0	29 (2.8)
	Self-employed	118 (14)	111 (10)	46 (6)	81 (7)	73 (3.3)	65 (6.3)
	Other	51 (6)	109 (9.9)	67 (9)	185 (16)	35 (1.6)	33 (3)

Personal Income/Month in RMB*	<700	46 (5.5)	178 (16)	471 (63)	840 (72)	329 (15)	283 (27)
	701–1500	506 (60)	710 (64)	258 (34)	304 (26)	1626 (74)	703 (67)
	>1501	291 (35)	227 (01)	20 (2.7)	16 (1.4)	243 (11)	64 (6.1)
	Median (IQR)	1100 (1200)	900 (600)	550 (500)	480 (400)	900 (250)	800 (250)

*US$1 = 8.2 Chinese Renminbi (RMB)

** Of the 34 factories involved 16 were textile, 6 foodstuff, 5 electrical, 4 steel, 3 chemical

### Smoking prevalence

This is shown by gender and residence in Table 2. Overall 54% of all men and 1.8% of all women were classified as current smokers. Among men current smoking was *least *common in migrants (51%) with 58% in urban workers and 64% in rural dwellers.

**Table 2:** Smoking prevalence by residence and gender. All numbers are percentages.

		Urban		Rural		Migrant	
		M	F	M	F	M	F
		n = 843	n = 1115	n = 749	n = 1160	n = 2198	n = 1050

Smoking behaviour	Never	27	97	28	98	36	97
	Have quit	8.7	0.5	7	0.3	9.5	0.4
	<6/week	6.2	1.3	1.1	0.2	4.0	1.0
	1–3/day	12	0.9	4.5	0.5	22	1.0
	4–9/day	16	0.6	10	0.3	11	0.4
	10–20/day	22	0.2	27	0.2	13	0.2
	>20/day	7.8	0.3	22	0.1	4.7	0
Smoking behaviour aggregates	Current	58	2.0	64	1.1	51	1.6
	Moderate/heavy	30	0.5	49	0.3	18	0.2

Table 3 shows patterns of current and moderate/heavy smoking by age and occupation in all three groups. The gap between rural, migrant and urban increased with moderate and heavy smoking, so the proportion of rural workers who smoked heavily was 4.7 times that of migrants. Forty-nine percent of all rural males smoke more than 10 cigarettes per day and 22% smoke more than 20. Few women smoked and very few smoked heavily. Prevalence of both current and moderate/heavy smoking increased with age in all three groups, but the trends were more marked for moderate/heavy smoking. Men over 40 were over twice as likely to be moderate/heavy smokers than men ages 21–30 in all three groups. The relatively low prevalence in the under-20 age group is explained partly by late uptake.

**Table 3:** Current and heavy smoking in male respondents by residence and age

		Urban n = 843	Rural n = 749	Migrant n = 2198
Current smoking by age	≤20	43	25	35
	21–30	64	60	50
	31–39	73	67	53
	≥40	65	64	59
Moderate/heavy smoking by age	≤20	15	15	5
	21–30	16	35	12
	31–39	37	54	25
	≥40	30	55	29
Current smoking by major occupation	Agriculture		74	
	Factory	53		48
	Construction	74		68
	Service	47		43
Moderate/heavy smoking by major occupation	Agriculture		31	
	Factory	17		11
	Construction	39		36
	Service	15		9

Among men construction and agricultural workers had the highest prevalence of current smoking (71% and 74% respectively) dropping to 51% in manufacturing and 45% in the service sector (hotels/bars and retail). Agricultural workers and construction workers were also over twice as likely to be moderate or heavy smokers: construction workers 38%, agricultural workers 31% compared with manufacturing 14% and service 12%.

The age of onset of smoking was predominantly in the late teens with rural dwellers starting earliest (90% starting under the age of 20, compared with 55% of urban workers). Lower education level was significantly associated with early onset of smoking (OR 2.4 CI 2.1,2.7 P < 0.001) and this relationship persisted after controlling for residence status. (OR 1.9 CI 1.6,2.1 P < 0.001).

Risk factors for both current and moderate/heavy smoking are shown in Table 4. Being married, having children, drinking alcohol regularly and higher personal income were all significantly associated with current smoking behaviour. Odds ratios for all of these variables increased for moderate and heavy smoking. Lower educational attainment was significantly associated with moderate and heavy smoking but not current smoking. However, after adjustment for age and residence, only rural living and regular drinking (at least once per week) were significantly associated with current smoking. In contrast the picture for moderate/heavy smoking shows all predictor variables as highly significant after adjustment for age and residence status. So rural residence, older age, lower education, drinking and higher income are all significantly associated with moderate/heavy smoking after adjustment.

**Table 4:** Risk factors for current and heavy smoking in men only: percentages, unadjusted and adjusted odds ratios with 95% confidence intervals.

		**Current smoking**					**Moderate/heavy**				
		**%**	**OR 95% CI**	**P-value**	Adjusted OR*	**P-value**	**%**	**OR 95% CI**	**P-value**	AdjustedOR *	**P-value**

**Residence**	**Urban**	58	1.13	0.009	1.17	0.005	18	1.78 (1.66, 1.9)	<0.0001	1.49	<0.0001
	**Rural**	64	1.01,1.2		1.1,1.3		50			1.29,1.75	
	**Migr't**	51					28				
**Age**	**<30**	55	1.27	<0.0001	1.12	0.08	12	4.2 (3.5,5.0)	<0.0001	3.82	<0.0001
	**>30**	61	1.1,1.4		0.98,1.2		38			3.24,4.52	
**Education**	**Low**	58	1.0	0.5	0.9	0.6	31	2.2 (2,2.5)	<0.0001	1.26	0.008
	**High**	59	0.9,1.2		0.8,1.1		17			1.05, 1.49	
**Marital status**	**Ever**	61	1.3	<0.0001	0.89	0.14	34	1.3 (1.2,1.4)	<0.0001	1.1	0.06
	**Never**	55	1.1,1.5		0.77,1.03		26			1.0,1.2	
**Children**	**Yes**	61	0.8	<0.0001	1.17	0.04	35	1.4 (1.2,1.6)	<0.0001	1.2	0.05
	**No**	54	0.7,0.9		1.0,1.36		24			1.06,1.2	
**Drinking**	**Yes**	68	0.44	<0.0001	0.14	<0.001	32	0.53 (0.4, 0.6)	<0.0001	0.2	<0.0001
	**No**	48	0.4,0.51		0.13,0.16		20			0.18,0.24	
**Personal Income RMB/month**	**<900**	55	1.3	<0.0001	1.2	0.07	20	1.3 (1.2,1.6)	<0.0001	1.8	<0.0001
	**>900**	61	1.1,1.5		1.0,1.3		26			1.5,2.1	

* model adjusted for age and residence only

### Smoking cessation

Nine percent (n = 333) of all male respondents had quit smoking for at least one month. There were small differences across the three groups: 8.7% of the urban workers, 7% of the rural groups and 9.5% of the migrants. However, on detailed questioning around half (52%) of these had only experimented with smoking and had never become regular smokers. Successful quitters were better educated (11% higher education level, 6% lower, P < 0.0001). The 333 respondents who had quit were asked why, and were allowed to select reasons (any number) from a list. The major reasons given were to prevent future illness (58%), current illness (31%), family pressures (20%) and financial considerations (20%). They were also specifically asked if anything had aided the quitting process: 12% mentioned a form of traditional Chinese medicine, but none mentioned nicotine replacement. Nearly half (46%) of the migrants and 21% of the urban workers who had quit stated that not being permitted to smoke in their workunit had helped them.

Current smokers (n = 2133) were all asked about attempts at quitting. Thirteen percent of current smokers had ever tried to quit, with 7% having tried in the last year, with urban workers almost twice as likely to have attempted to quit (18%) than the rural workers (10%) or migrants (9.4%). Higher proportions said they intended to quit at some point with migrants most likely to intend to quit: 28% of the migrants, 22% of the urban workers but just 14% of the rural workers. Of these (n = 524) over half (58%) thought it would be very difficult and were not optimistic about success. When asked about what forms of help were available 27% mentioned medicine, (only 5% specifically nicotine replacement) and 15% telephone helplines. Most (91%) said that willpower was the most important factor.

### Knowledge

It is thought that one of the reasons the poor and less well-educated are more prone to smoking is because of their lack of awareness of the health risks [1]. The overwhelming majority of the sample (94%) were aware that smoking was bad for health with no significant difference between smokers (96%) and non-smokers(92%) (P = 0.1) Most could also name illnesses which could be attributable to smoking: 74% named cancer and 63% lung/breathing problems. Few (12%) named cardiovascular conditions. There was much less awareness of the risks of passive smoking with only 25% of the whole sample mentioning it even when prompted.

### Expenditure on smoking

Mean absolute expenditure on cigarettes was 117 RMB per month, SD 126, median 80, interquartile range 90. (see Table 5) There were significant differences across the three groups: the median for migrants was 60 RMB (interquartile range 80), rural median 100 RMB, (IQR 100) and the urban median was 200 RMB (IQR 230, P < 0.0001). Despite rural workers being the heaviest smokers, their median expenditure was half that of urban workers. We calculated a mean price per cigarette for the three groups. Rural workers smoked cigarettes costing an average of 0.21 RMB per cigarette, similar to the price paid by migrants, 0. 26 RMB but much less than that of urban dwellers at 0.59 per cigarette. There was no significant difference between numbers of cigarettes consumed between lower and higher income individuals across the three groups. Odds ratios for moderate smoking for higher vs lower income individuals were 1.1 CI 1.0,1.3 (P = 0.06) and for heavy 1.0, CI 0.9,1.1 (P = 0.8). Expenditure as a proportion of personal income shows that an overall mean of 11% of personal income is spent on smoking (range 0 to 38%) with significant differences between the three populations: migrants 9.4%, urban workers 13.5%. and rural residents 15.4% (P < 0.0001).

**Table 5:** Smoking behaviour and expenditure of current male smokers (n = 2233)

		Urban n = 539	Rural n = 486	Migrant n = 1208
Age of uptake of smoking in years	<15	3.4	5.6	3.5
	15–20	52	85	74
	21–25	37	10	15
	>25	8	0	3.0
Quitting	Have tried to quit in last year	9.1	3.0	7.1
	Ever tried to quit	18	10	9.4
	Intend to quit	22	14	28
Smoking expenditure RMB/month	<20	4.6	2.3	9.7
	20–49	11	17	31
	50–99	11	28	25
	100–249	45	43	30
	250+	28	9.6	4.9
	Median (IQR)	200 (230)	100 (100)	60(80)
Smoking expenditure as % of personal income		13.5	15.4	9.4

We asked further exploratory questions, of moderate and heavy smokers only, (n = 616) about the impact that smoking had on other personal and household expenditures. Of the rural moderate/heavy smokers 75% admitted that expenditure on smoking affected their ability to pay for other things, compared with (45%) of the urban workers and (61%) of the migrants. Respondents were asked what types of expenditure they felt were most affected and they were asked to select from a list of options. The three major specific expenditures cited were health care (51%), savings (45%) and purchase of large household items(42%).

## Discussion

The study highlights a number of important issues which have relevance for tobacco control measures. First, the prevalence of smoking in an important group of Chinese men remains high. Interpretation of trends is hampered by differing definitions of current smoking across studies and the different population groups studied but some trends do emerge. Data from the 1996 national prevalence study [20] classified 67% of Zhejiang men as current smokers, (using the same definition as ours) compared with our estimate of 54%. Two recent smaller studies carried-out in Hangzhou residents found rates for smoking among adult males to be 47% [22] and 43% [23] respectively. Clearly direct comparison between these studies is hampered by different definitions of current smoking, different samples used and the fact that we sampled only in low-income workers who would be expected to smoke more. Given that these figures seem to be consistently lower across more recent studies indicates that smoking prevalence in male Chinese *may *be decreasing and at the very least is not on the increase. This would concur with findings from other studies. Cheng found 57% of Chinese male peasants across five provinces were current smokers, which was described as lower than had been reported previously[4], and Yang found a 1.8% reduction in ever smoking between 1996 and 2002 in men across 145 sentinel surveillance sites[3]. Trends across age cohorts in our study also tend to support this view. The low prevalence of smoking in the under 20 cohort relates largely to late uptake, as illustrated by responses to the direct question about age of onset of smoking and which is has been shown in other studies. [9,20,24]. But for the other three age groups there is an upward trend in smoking prevalence, which is more marked for moderate and heavy smoking. This suggests that younger age groups are taking-up smoking less or are quitting more. Since relatively small numbers have quit the former is a more plausible explanation.

Our *a priori *hypothesis that migrants would be heavy smokers and would warrant intensive tobacco control measures has been refuted: migrants take up smoking later, are least likely to become regular or heavy smokers, and are more likely to want to quit, and to be successful when they do. Another study in rural-urban migrants in Beijing reported male current smoking prevalence of 52%, though there were no comparison groups [25]. These lower than expected figures may relate in part to the fact that migrants leave home in the first place in order to make money [15] and so are highly motivated to cut down or quit smoking in order to save more money to take back to their rural communities.

More worrying are the high rates of heavy smoking, especially in the rural population, where nearly half the men (49%) smoked more than 10 cigarettes per day and 22% smoked more than 20 cigarettes per day suggesting that the burden of disease attributable to smoking in eastern rural China is huge. Given that the rural dwellers are the poorest group and access to healthcare is compromised by high expenditures on cigarettes, it is clear that tobacco control measures should do far more to target rural populations.

There is also an indication that numbers of quitters, though small, may be on the increase. The 1996 study reported 3.5% successful and 15% intending quitters in Zhejiang [19] compared with 7% and 22% respectively in our study. Again it must be noted that comparison is hampered by the different samples for the two studies. However, this does suggest that quitting may be on the increase. In a recent Hangzhou-based study of male smokers 22% claimed to have tried to quit, though quitting was not clearly defined [22]. In this study social stigma was given as one of the reasons for wanting to quit by one quarter of the sample. In our study no-one gave social stigma as a reason, suggesting that at least among low income groups smoking is not stigmatised and indicating that health promotion measures should perhaps focus on trying to make smoking less socially acceptable. The data on quitting has important implications for tobacco control measures. With 22% of men stating their intention to quit and most acknowledging how difficult quitting is, support should be made available for them. There is good evidence from a number of countries that nicotine replacement therapy (patches, gum etc) can increase success rates [26]. These therapies were virtually unknown to the respondents in this study, but they could be produced cheaply and be made widely available. Again it was noted that workplace bans had helped some of those who had quit, suggesting that bans on smoking in workplaces and public places should be broadened and enforced. In Zhejiang enforcement measures especially in the workplace have been weak. It was also notable that we found that where smoking was permitted, in the agricultural and construction sectors, there were particularly high rates of heavy smoking, compared with those workunits where there are restrictions such as in factories, shops and hotels Smoking restrictions elsewhere have been shown to help both with quitting and with reducing numbers of cigarettes smoked [27,9].

We show that expenditure on smoking constitutes a large proportion of personal income, an average of 11%, but rising to 15.4% for rural inhabitants. The opportunity costs are considerable with disproportionate effects in poor rural households, including compromising access to healthcare. Other studies have calculated expenditure on smoking as a percentage of household income, producing lower estimates, because there is usually one smoker per couple [30]. The findings indicate that information about the opportunity costs of smoking could be used in health promotion messages, to discourage youngsters from starting and to promote cessation efforts. Our findings on expenditure also raise questions about the role of tobacco taxation as deterrent in low income groups. In 1997 tobacco taxation accounted for 11% of all central government revenue [31]. The cost of cigarettes varies from $0.5 to around $8 per pack in China, of which around 40% is tax [32]. With such cheap cigarettes available cost clearly becomes less of a deterrent. This is shown in our study by the finding that the heaviest smokers were the poor rural inhabitants; the poor just smoke cheaper cigarettes. But price elasticity for cigarettes (the reduction in consumption which follows an increase in unit price) in China has been a matter for debate [32,33], and it is not clear to what degree individuals would reduce their consumption if prices were to rise, and whether increased taxation would simply disproportionately punish the poor, given evidence that the poor are less likely to quit than the wealthier [1,2]. This is one of the reasons the Chinese government has given for not using tougher fiscal measures [34].

Knowledge of the health risks of smoking was generally good, while knowledge of the dangers of passive smoking was poor. This is of particular concern given the finding that married and having children were positively associated with moderate and heavy smoking. Campaigns to raise awareness of the dangers of passive smoking should be incorporated into health promotion programmes.

The study has a number of limitations. First, this study was carried-out in low income working individuals in a rich eastern coastal province. While some inferences can be drawn from this sample to individuals of similar socioeconomic status in other coastal provinces, our sample does not try to be representative of other parts of China, especially the poor inland and Western provinces. Second, the study sampled workunits in the formal sector and did not include the self-employed, such as hawkers and street-traders, but it is estimated that these account for less than 5% of migrants in Hangzhou, so our sample is representative of the overwhelming majority. Third, the validity of self-report may be questionable, perhaps more so among groups for whom smoking may be less acceptable, such as women. It is also possible that the different situations in which the questionnaires were completed, workunits in the urban area and home in the rural area could present a source of bias. Fourth, comparisons of personal income across the three groups are crude, because of different purchasing power and lifestyle in rural areas. Finally, exploratory questions were only asked of moderate and heavy smokers, because they are most likely to incur opportunity costs, but this may limit the applicability of this section of the results.

## Conclusion

The results of this study have provided important pointers for tobacco control measures. Given the high rates of smoking among rural workers, these populations need to be specifically targeted for tobacco control measures. Up until now health promotion efforts in Zhejiang and other Chinese provinces have focused on urban areas with very limited (if any) activity in rural areas. Smoking restrictions in workplaces and public places should be widened and enforced. At present these are piecemeal and often not enforced. Support should be provided to smokers who want to quit and cheap aids to quitting should be made available. Although health education messages have had disappointing results in many countries [35] our study highlights areas which might serve as foci for health promotion messages: the dangers of passive smoking, the opportunity costs of smoking and making smoking less socially acceptable. In September 2005 China ratified the Framework Convention on Tobacco Control. This has created new impetus for tobacco control measures. This together with somewhat encouraging trends in prevalence and quitting, indicate that China may see further declines in smoking in the near future.

## Competing interests

The author(s) declare that they have no competing interests.

## Authors' contributions

TH and LL designed the study and developed the tools. YXJ and WHM supervised the data collection, data entry and analysis. TH wrote the paper to which all authors contributed. All authors have read and approved the final manuscript.

## Pre-publication history

The pre-publication history for this paper can be accessed here:


